# Supplementary figures and images for: Mycobacterial infection-induced miR-206 inhibits protective neutrophil recruitment via the CXCL12/CXCR4 signalling axis
Source: PLoS Pathog. 2021 Apr 7;17(4):e1009186. doi: 10.1371/journal.ppat.1009186 (PMC8055004; doi:10.1371/journal.ppat.1009186)

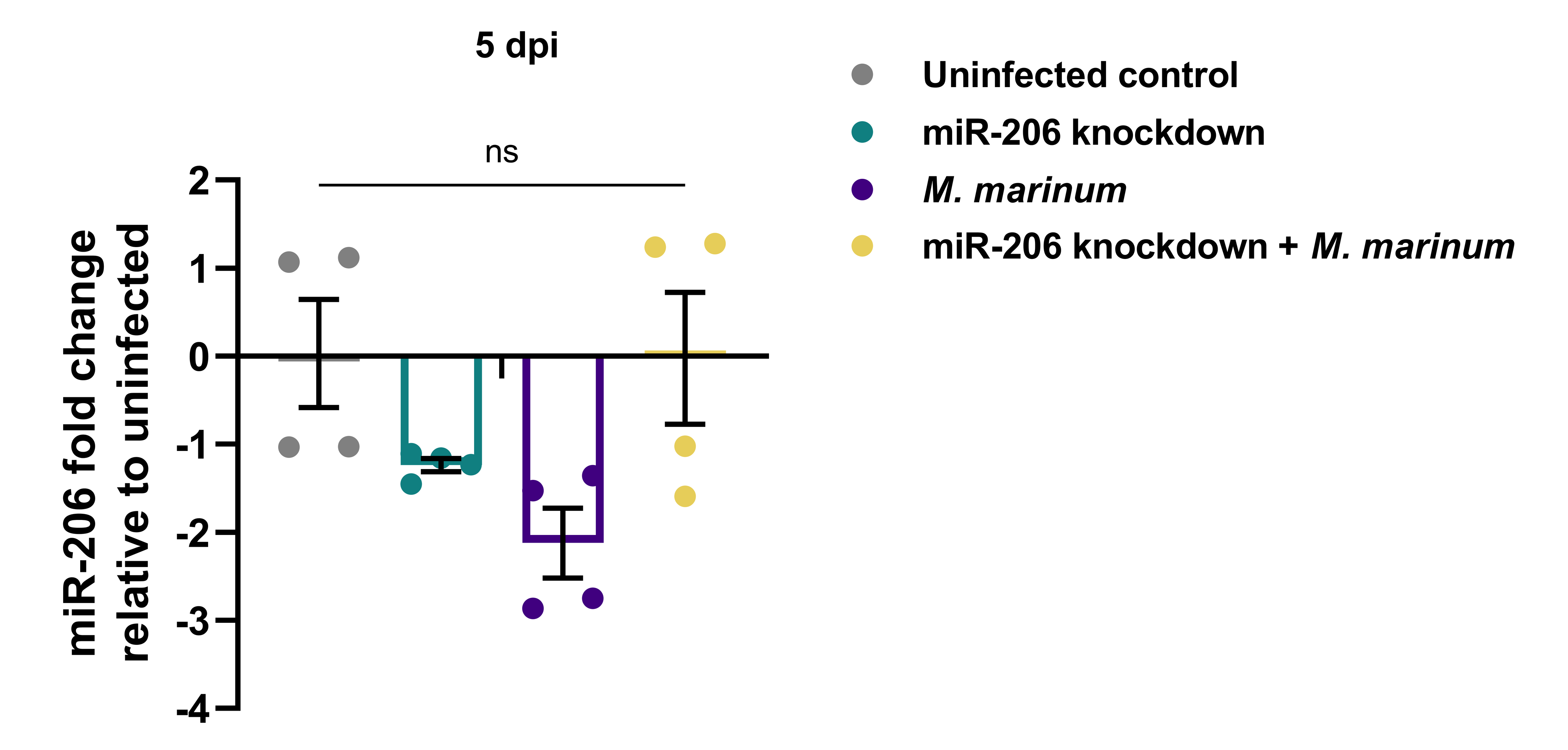

Supplement: S1 Fig — Expression of miR-206 was analysed by qPCR at 5 dpi following antagomiR knockdown. Each data point represents a single measurement of 10 pooled embryos and 2 biological replicates, with the mean and SEM shown. (TIF) [file ppat.1009186.s001.tif]

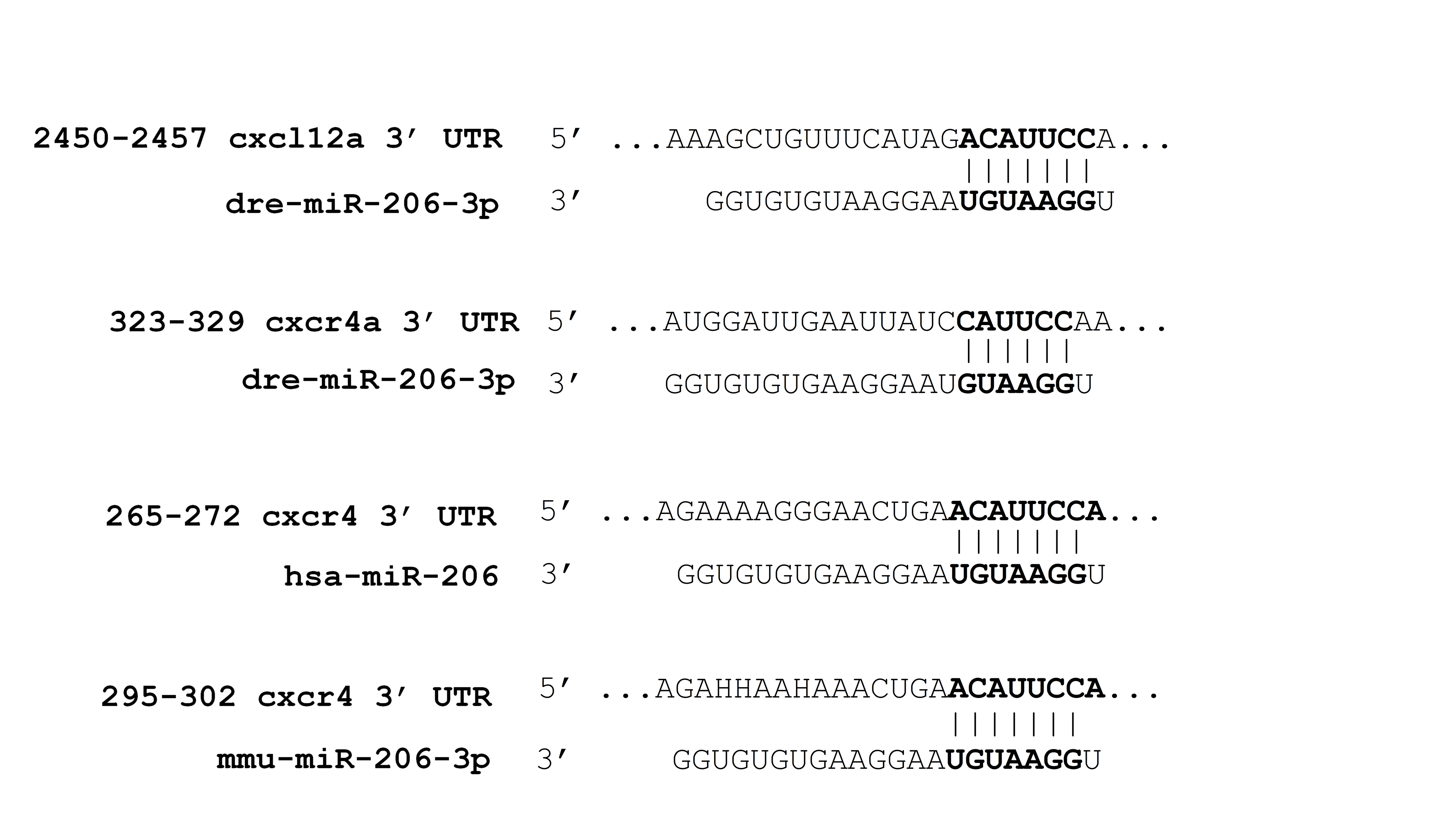

Supplement: S2 Fig — Alignment of potential target gene binding sites to miR-206 seed sequence predicted by TargetScan. (TIF) [file ppat.1009186.s002.tif]

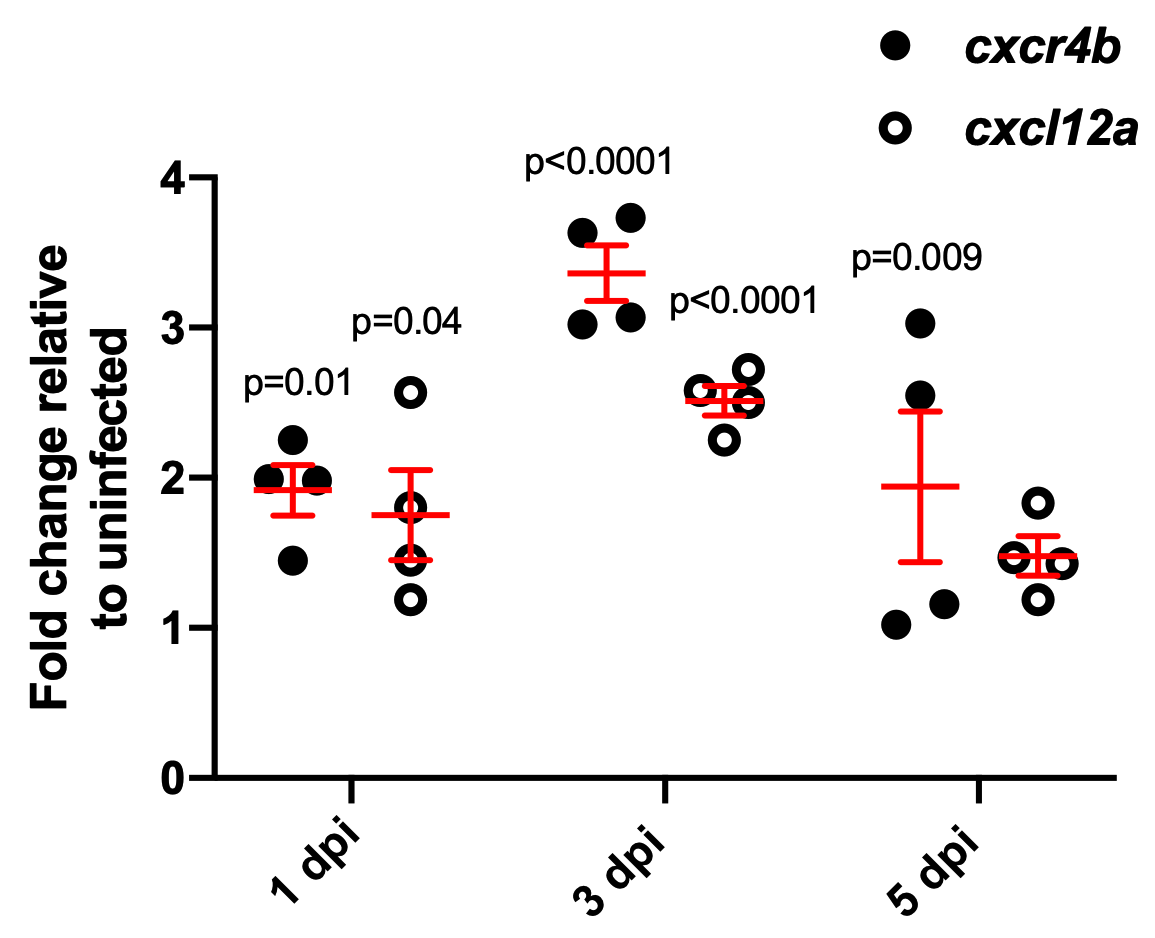

Supplement: S3 Fig — cxcr4b and cxcl12a transcript abundance was measured by qPCR in M. marinum infected embryos at 1, 3 and 5 dpi by qPCR. Each data point represents 10 embryos and contains 2 biological replicates. (TIFF) [file ppat.1009186.s003.tiff]

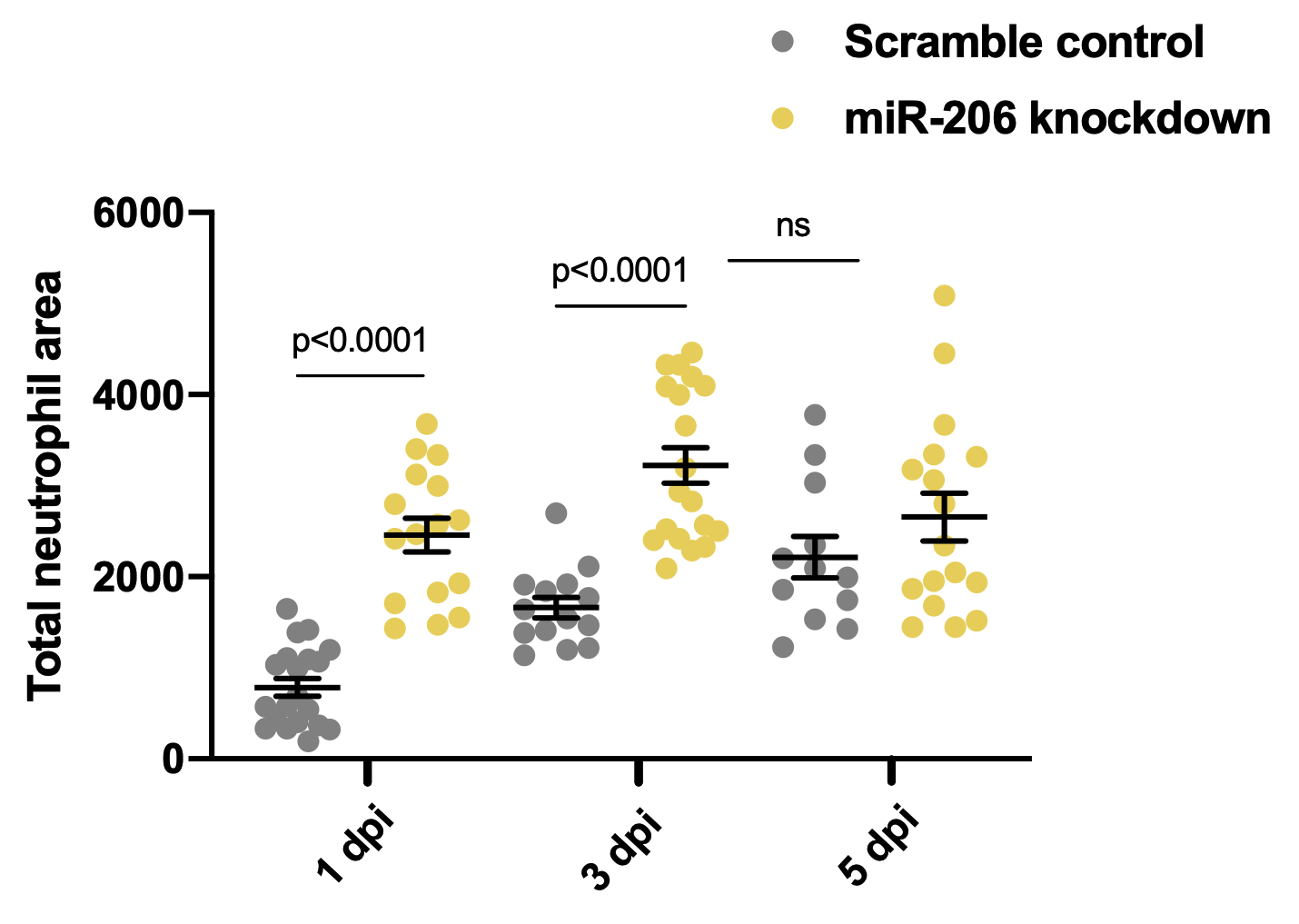

Supplement: S4 Fig — Whole-body neutrophil fluorescence at 1, 3, and 5 dpi in control and miR-206 knockdown embryos. Each data point represents a single neutrophil with the mean and SEM shown. (TIFF) [file ppat.1009186.s004.tiff]

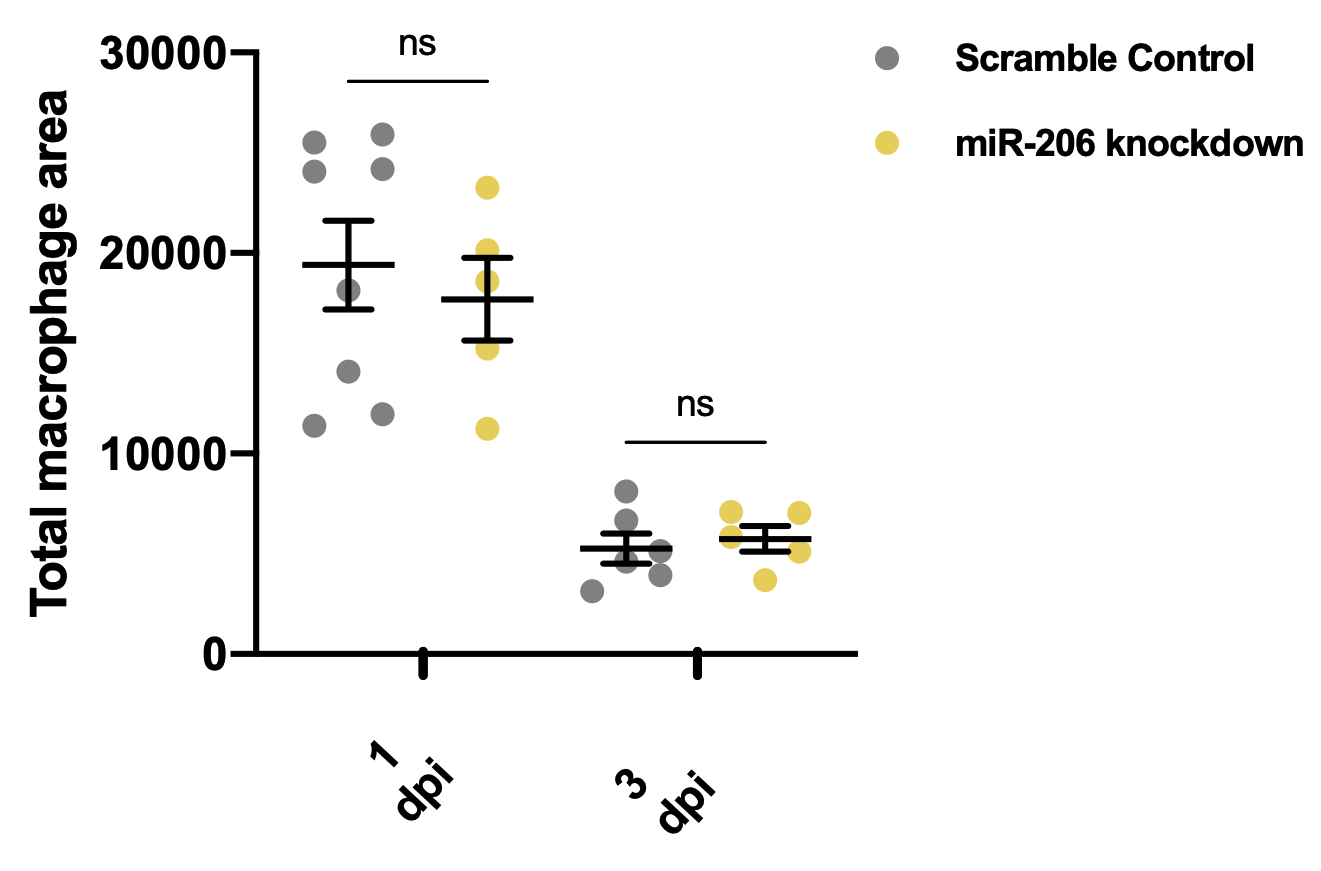

Supplement: S5 Fig — Following trunk infection with M. marinum, total macrophage fluorescence was measured at sites of infection. Each data point represents a single embryo with the mean and SEM shown. (TIFF) [file ppat.1009186.s005.tiff]

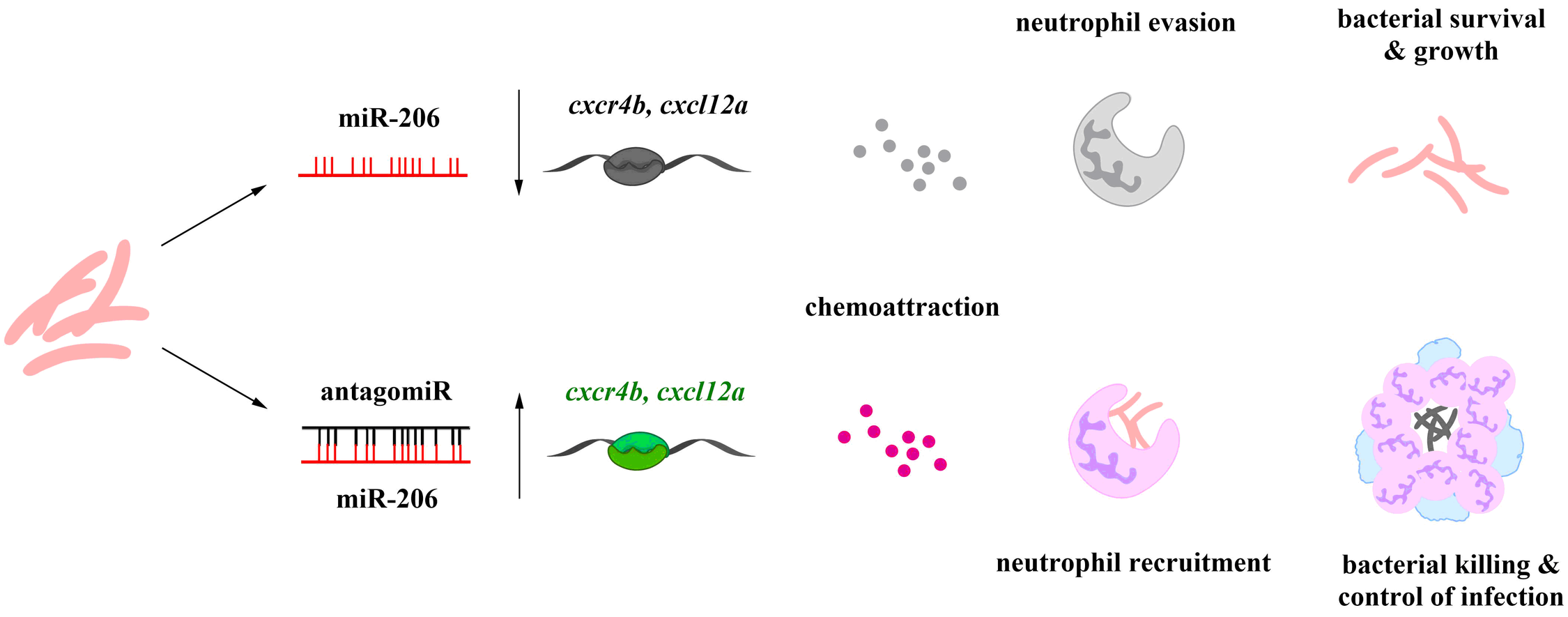

Supplement: S6 Fig — Top line: M. marinum infection induces the expression of host miR-206. miR-206 suppresses the expression of cxcr4b and cxcl12a which results in suboptimal neutrophil recruitment and supports bacterial growth. Bottom line: AntagmiR-mediated neutralisation of miR-206 allows increased expression of cxcr4b and cxcl12a which increases neutrophil recruitment and suppresses bacterial growth. (TIF) [file ppat.1009186.s006.tif]
