# Supplementary material for: Mycobacterial infection-induced miR-206 inhibits protective neutrophil recruitment via the CXCL12/CXCR4 signalling axis
Source: PLoS Pathog. 2021 Apr 7;17(4):e1009186. doi: 10.1371/journal.ppat.1009186 (PMC8055004; doi:10.1371/journal.ppat.1009186)
Supplement: Attachment — Submitted filename: PloS pathogens review_0107.pdf [file ppat.1009186.s013.pdf]

In this manuscript, Kathryn Wright et al. have used the zebrafish model of *Mycobacterium marinum* (M.m) infection and found that M.m induces a miRNA, miR-206 during infection of the embryos. They investigated the role of miR206 in the early stages of granuloma formation by challenging zebrafish embryos with the bacterium. The authors found that miR-206 upregulation coincides with more bacterial foci and knocking down miR206 with antagomir- mediated knockdown decreased bacterial burden. To identify the host targets of miR-206, they checked the expression of a chemokine and chemokine receptor (by using a target prediction algorithm, though the results of this analysis was not presented!) and demonstrated that miR-206 knock down leads to upregulation of Cxcr4a/b, elmo1, Cxcl12 during M.m infection. Upregulation of the CXCR4/CXCL12 genes following miR-206 knock down resulted in an increased neutrophil response at the granulomatous foci and associated with reduction in bacterial foci. Wright et al. then used two different strategies (gene knock down by CrispR/Cas9 and pharmacological inhibition of Cxcr4 pathway with the selective inhibitor Plerixafor/AMD3100 to show that inhibiting the CXCR4/CXCL2 signaling pathway blunts the neutrophil response at the infection site, that ultimately result in high bacterial burden. Based on these results, the authors propose that M.m infection induces miR-206 expression to prevent a protective neutrophil response early during granuloma formation that might lead to a better outcome. The manuscript is well written, and experiments are well controlled. The identification of miR-206 mediated targeting of cxcr4/cxcl12 signaling and downstream neutrophil recruitment adds to the current understanding of protective innate immunity to mycobacterial infections. However, few concerns remain that needs the author's attention.

1. Whether an augmented neutrophil influx after miR-206 knock down directly causing the bacterial killing was not investigated.
2. The authors did not show the effect of miR-206 knock down on other immune cells especially macrophages and monocytes that may play a major antimicrobial role.
3. Since neutrophils were shown to be pathological during TB in multiple models of murine TB and humans, does the excessive infiltration of neutrophils early after miR-206 knock down affect granuloma fate/outcome at later time points? The neutrophil response was investigated up to 3dpi.
4. Since miR-206 expression declines after 3 dpi, what happens to the expression level of CXCR4 and CXCL12 ? This data would add value to the existing results. What is the effect of declining miR-206 expression on neutrophil response and bacterial burden at 5dpi? These data are important in making the conclusion the authors have made.
5. CXCR4/CXCL12 is needed to retain neutrophils in the bone marrow of mammals and perturbation of this signaling is a requisite for neutrophil mobilization. The high neutrophil response in the cxcr4 and cxcl12 kd embryos suggest that the neutrophils are retained at the site of infection. Are these neutrophils newly

recruited cells or ageing cells that are retained at the site of infection? Moreover, cxcr2 has been shown to regulate neutrophil influx during mycobacterial infection (Dorhoi et al, 2013; Lovewell et al. 2020). Was CXCR2 expression checked in this study or filtered by the algorithm? The authors need to comment on this.

Minor Comments:

1. Fig.1a. The fold changes should be expressed as relative to uninfected.
2. Fig. 1f. the x-axis should read as 6h and 1dpi as mentioned in the text and figure legend.
3. The authors must comment on the results of the predicted gene targets of miR-206 as checked in the figure 2 though the authors cited the original publications describing this bioinformatic target prediction algorithms (line 138)
4. The miR-206 and cxcr4b or cxcl12a DKD embryos had significantly fewer bacterial areas than Cxcr4b or cxcl12a KD embryos suggesting that other pathways might regulate bacterial control at granuloma foci. The authors suggested in addition to cxcr4/cxcl12 signaling, other pathways might be involved. The authors should discuss these putative pathways (though they mention about elmo1 which regulates cell motility) that are targets of miR-206.
