# Supplementary material for: Mycobacterial infection-induced miR-206 inhibits protective neutrophil recruitment via the CXCL12/CXCR4 signalling axis
Source: PLoS Pathog. 2021 Apr 7;17(4):e1009186. doi: 10.1371/journal.ppat.1009186 (PMC8055004; doi:10.1371/journal.ppat.1009186)
Supplement: Attachment — Submitted filename: Response to reviewers.docx [file ppat.1009186.s014.docx]

**Response to reviewers**

**Major Issues: Key Experiments Required for Acceptance**

1. Whether an augmented neutrophil influx after miR-206 knock down directly causing the bacterial killing was not investigated.

**Direct neutrophil and *M. marinum* interactions were observed at 5 hour intervals from 1 to 15 hpi (Figure 4, reproduced below). miR-206 knockdown produced consistently smaller granulomas than control infected embryos as assessed by bacterial burden (Figure 4A-C). This containment of bacterial growth coincided with increased neutrophil recruitment to, and retention at, sites of infection as measured by the colocalization of bacterial and neutrophil fluorescence (Figure 4D-E). Text related to Figure 4 begin around Line 238 in the unmarked manuscript.**

**We note that this is not a direct measure of the intracellular killing of bacteria by neutrophils as reported in other *in vivo* zebrafish manuscripts. We do not have access to the high-end light sheet or confocal microscopes required for these analyses. While other factors are likely to contribute to the control of infection in miR-206 knockdown, as there is a normal macrophage response, we believe the increased influx and retention of neutrophils is clearly an important mechanism.**

2. The authors did not show the effect of miR-206 knock down on other immune cells especially macrophages and monocytes that may play a major antimicrobial role.

**Macrophage numbers following trunk infection was assessed at 1 and 3 dpi. No difference between the fluorescent area of mfap4-expressing macrophages at granulomas between control and miR-206 knockdown embryos was observed. Data has been included as Supplementary figure 4 (reproduced below) and text from Line 217.**

3. Since neutrophils were shown to be pathological during TB in multiple models of murine TB and humans, does the excessive infiltration of neutrophils early after miR-206 knock down affect granuloma fate/outcome at later time points? The neutrophil response was investigated up to 3dpi.

**We have found the antagomiR effectiveness to be reduced past 3 dpi/4-5 dpf, as such it was not possible to reliably examine later timepoints. We acknowledge that this is a limitation of the transient knockdown system and believe a full knockout model would be better suited to investigate later timepoints and the immunopathology of an excessive neutrophil response. We have included the figure below as a supplementary figure to highlight the limitations of antagomiR knockdown during infection past 3 dpi. The data suggests the antagomiR is partially effective in unchallenged embryos but fails to further reduce miR-206 levels in infected embryos, thus demonstrating lack of reagent effectiveness at this later timepoint.**

**Conceptually, we note that increased early neutrophil recruitment has been shown to be protective (eg Yang et al 2012, Elks et al 2013, Cronan et al 2016), these references have been added to line 332.**

4. Since miR-206 expression declines after 3 dpi, what happens to the expression level of CXCR4 and CXCL12? This data would add value to the existing results. What is the effect of declining miR-206 expression on neutrophil response and bacterial burden at 5dpi? These data are important in making the conclusion the authors have made.

**We have analysed the expression of *cxcr4* and *cxcl12* at 1, 3, 5 dpi in *M. marinum* infected embryos. Expression of these genes is consistently upregulated across 1-5 dpi and there do not appear to be further increases in expression correlating with decreasing miR-206 in WT infections (Supplementary Figure 3A, reproduced below). We believe it is difficult to delineate between miR-206-dependent expression and general infection-induced expression. As our antagomiR knockdown methodology is not efficacious at 5 dpi in *M. marinum* infection (see above), we were unable to further explore the neutrophil/Cxcr4/Cxcl12/miR-206 axis past 3 dpi.**

5. CXCR4/CXCL12 signaling is essential in retaining neutrophils in the bone marrow of mammals and perturbation of this signaling is a requisite for neutrophil mobilization. The high neutrophil response in the cxcr4 and cxcl12 kd zebrafish embryos suggest that the neutrophils are retained at the site of infection. Are these neutrophils newly recruited cells or ageing cells that are retained at the site of infection?

**We have included a new analysis of our imaging data in Figure 3D (reproduced below)showing increased retention of individual neutrophils at the site of infection. This demonstrates that the increase in neutrophil numbers is caused by both additional recruitment and retention.**

Moreover, CXCR2 has been shown to regulate neutrophil influx during mycobacterial infection (Dorhoi etal, 2013; Lovewell et al. 2020). Was CXCR2 expression checked in this study or filtered by the algorithm? The authors need to comment on this.

**CXCR2 was not identified as a potential target from the prediction algorithm (Supplementary File 1) and expression was not measured. Pathway interaction is discussed in Lines 396-401**

Reviewer #2: The writing in the paper is generally clear and concise, but the authors do not provide enough background in the introduction to fully appreciate their work and the discussion could use a little more depth. More detail is needed on the zebrafish model, mRNAs in TB/mycobacterial infections, etc.

**The introduction has been expanded to include greater background on the zebrafish-*M. marinum* model (Lines 82-94) and the role of miRNA in mycobacterial infections (Lines 65-72).**

**Discussion expanded, also in response to Reviewer 1 minor comment 4 requesting more discussion of putative pathways that could interact or complement the CXCR4/CXCL12 pathway that we have focused on (starting Line 355).**

The materials and methods section needs more detail than is currently provided in several areas so the reader doesn’t have to go back to other papers to find out important details (e.g. how bacteria were cultured and quantified, etc).

**We have expanded the methods section 5 to elaborate on our bacterial culture and quantification methods. Section 12 has been expanded to elaborate on our use of fluorescent pixel count.**

This may also help with questions I had but weren’t addressed in the paper (e.g. why were the timepoints selected, why were neutrophils targeted, etc). It was also unclear why the CXCR4/CXCL12 pathway was selected in their analysis rather than the other genes they profiled (Fig. 2). These genes are briefly mentioned these other genes in the discussion but are not well integrated well into the story the authors are telling.

**Discussion expanded, also in response to Reviewer 1 minor comment 4 requesting more discussion of putative pathways that could interact or complement the CXCR4/CXCL12 pathway that we have focused on.**

The paper might also benefit from having a model showing how the author’s link miR-206, downregulated CXCR4/CXCL12, and decreased neutrophil infiltration with reduced bacterial control.

**We have added a graphical abstract style summary diagram that is referenced at the start of the discussion section.**

Reviewer #3: No new experiments requested.

**Minor Issues: Editorial and Data Presentation Modifications**
Reviewer #1: Minor Comments:
1. Fig.1a. The fold changes should be expressed as relative to uninfected.

**We have adjusted the Y axis labels on Figures 1ABD and 2 ABCD as appropriate.**

2. Fig. 1f. the x-axis should read as 6h and 1dpi as mentioned in the text and figure legend.

**We have corrected this x-axis label.**

3. The authors must comment on the results of the predicted gene targets of miR-206 as checked in the figure 2 though the authors cited the original publications describing this bioinformatic target prediction algorithms (line 138)

**We have provided our output target lists as a new Supplementary File 1.**

4. The miR-206 and cxcr4b or cxcl12a DKD embryos had significantly fewer bacterial areas than Cxcr4b or cxcl12a KD embryos suggesting that other pathways might regulate bacterial control at granuloma foci. The authors suggested in addition to cxcr4/cxcl12 signaling, other pathways might be involved. The authors should discuss these putative pathways (though they mention about elmo1 which regulates cell motility) that are targets of miR-206.

**We agree that it is likely other pathways are also co-regulating and controlling bacteria. We have added to our discussion of the putative pathways in the discussion section.**

Reviewer #2:

- Protein nomenclature for CXCR4/CXCL12/VEGF/MMP9/TIMP3 should be capitalized.

**We have capitalised the instances of these when used in a non-zebrafish context. We note that when referring to our data, rather than published work using other species, we have maintained the Protein and *gene* formatting used for zebrafish.**

- CRISPR should be capitalized.

**We have capitalised all instances of CRISPR.**

- ‘MicroRNA’ line 42 and 56 should be capitalized.

**We have capitalised these instances of MicroRNA.**

- ‘gene silencing’ doesn’t need quotation marks

**We have removed quotation marks.**

- THP-1s would be better described as a monocyte-like cell line rather than as leukocytic cells

**We have replaced “leukocytic” with “monocyte-like”**

Reviewer #3: None
